# Genetic-Based Susceptibility of a Foundation Tree to Herbivory Interacts With Climate to Influence Arthropod Community Composition, Diversity, and Resilience

**DOI:** 10.3389/fpls.2018.01831

**Published:** 2018-12-11

**Authors:** Adrian C. Stone, Catherine A. Gehring, Neil S. Cobb, Thomas G. Whitham

**Affiliations:** ^1^Department of Biology, Metropolitan State University, Denver, CO, United States; ^2^Department of Biological Sciences, Northern Arizona University, Flagstaff, AZ, United States; ^3^Merriam-Powell Center for Environmental Research, Flagstaff, AZ, United States

**Keywords:** pinyon pine, pinyon needle scale, drought, climate change, arthropod community, community resilience, direct and indirect genetic effects, herbivore resistance and susceptibility

## Abstract

Understanding how genetic-based traits of plants interact with climate to affect associated communities will help improve predictions of climate change impacts on biodiversity. However, few community-level studies have addressed such interactions. Pinyon pine (*Pinus edulis*) in the southwestern U.S. shows genetic-based resistance and susceptibility to pinyon needle scale (*Matsucoccus acalyptus*). We sought to determine if susceptibility to scale herbivory influenced the diversity and composition of the extended community of 250+ arthropod species, and if this influence would be consistent across consecutive years, an extreme drought year followed by a moderate drought year. Because scale insects alter the architecture of susceptible trees, it is difficult to separate the direct influences of susceptibility on arthropod communities from the indirect influences of scale-altered tree architecture. To separate these influences, scales were experimentally excluded from susceptible trees for 15 years creating susceptible trees with the architecture of resistant trees, hereafter referred to as scale-excluded trees. Five patterns emerged. (1) In both years, arthropod abundance was 3-4X lower on susceptible trees compared to resistant and scale-excluded trees. (2) Species accumulation curves show that alpha and gamma diversity were 2-3X lower on susceptible trees compared to resistant and scale-excluded trees. (3) Reaction norms of arthropod richness and abundance on individual tree genotypes across years showed genotypic variation in the community response to changes in climate. (4) The genetic-based influence of susceptibility on arthropod community composition is climate dependent. During extreme drought, community composition on scale-excluded trees resembled susceptible trees indicating composition was strongly influenced by tree genetics independent of tree architecture. However, under moderate drought, community composition on scale-excluded trees resembled resistant trees indicating traits associated with tree architecture became more important. (5) One year after extreme drought, the arthropod community rebounded sharply. However, there was a much greater rebound in richness and abundance on resistant compared to susceptible trees suggesting that reduced resiliency in the arthropod community is associated with susceptibility. These results argue that individual genetic-based plant-herbivore interactions can directly and indirectly impact community-level diversity, which is modulated by climate. Understanding such interactions is important for assessing the impacts of climate change on biodiversity.

## Introduction

Despite a growing understanding of the importance of intra-specific genetic variation within foundation plant species in structuring their associated communities (Whitham et al., 2006; Des Roches et al., 2018; Koricheva and Hayes, 2018), few studies have incorporated both genetics and climate when quantifying these interactions (Johnson and Agrawal, 2005; Sthultz et al., 2009b; Gehring et al., 2017). Through direct and indirect genetic-based influences, foundation species have the potential to have impacts on biodiversity at many trophic levels as well as on ecosystem scale processes (reviews by Ellison et al., 2005; Whitham et al., 2012; Crutsinger, 2016). Furthermore, climate change is likely to have major consequences for the interactions among foundation species and their dependent communities (Parmesan and Yohe, 2003; Whitham et al., 2006; Gilman et al., 2010; Stone et al., 2010; Nooten et al., 2014). Taking a genetic-based approach allows researchers to place the impacts of climate change within an evolutionary framework (e.g., Grant and Grant, 2002; Franks et al., 2007; Aitken et al., 2008; O’Neill et al., 2008; Sthultz et al., 2009a; Grady et al., 2011, 2015; Busby et al., 2014; Gehring et al., 2017).

Foundation tree species influence the structure of associated communities in several ways. There are direct interactions with herbivores or species that use the trees for habitat that are often mediated by plant architecture and chemistry, and there are indirect influences that extend over many trophic levels as the species that are directly associated with the trees interact with other trophic levels or physically alter the plant. Genetic influences that modulate the direct interactions have the potential to cascade up to higher trophic levels and indirectly influence extended community structure (Bolnick et al., 2011; Bailey et al., 2014). These influences are referred to as interspecific indirect genetic effects (IIGEs; Shuster et al., 2006). Often these indirect interactions between plants and higher trophic levels come from plant modifications induced by herbivores because the effects of herbivory persist after initial herbivore activity has ended (Jones et al., 1994).

Herbivore–plant interactions can be genotype dependent (Crawford et al., 2007) or contingent on a plant’s resistance or susceptibility to specific herbivores, which often have a genetic basis (Dickson and Whitham, 1996; Gehring et al., 1997; Agrawal, 2004; Keith et al., 2017). Most studies on plant mediated indirect effects have focused on interactions of just a few species or trophic groups, while interactions between foundation species (e.g., a foundation herbivore and its host plant) that may indirectly affect a much larger associated community have been less studied (Ellison et al., 2005; Bailey and Whitham, 2007; Keith et al., 2010; Busby et al., 2015). Furthermore, few studies have addressed this issue in the context of genetic-based resistance and susceptibility to a foundation herbivore that physically alters the architecture of its host plant (but see Sthultz et al., 2009b). Quantifying whole community responses to plant mediated indirect effects is important because it represents the end result of multiple species interactions that can be difficult to isolate but are important for understanding community structure (Bangert et al., 2006), and the underlying mechanisms that maintain biodiversity (Ohgushi, 2005).

**FIGURE 1:**
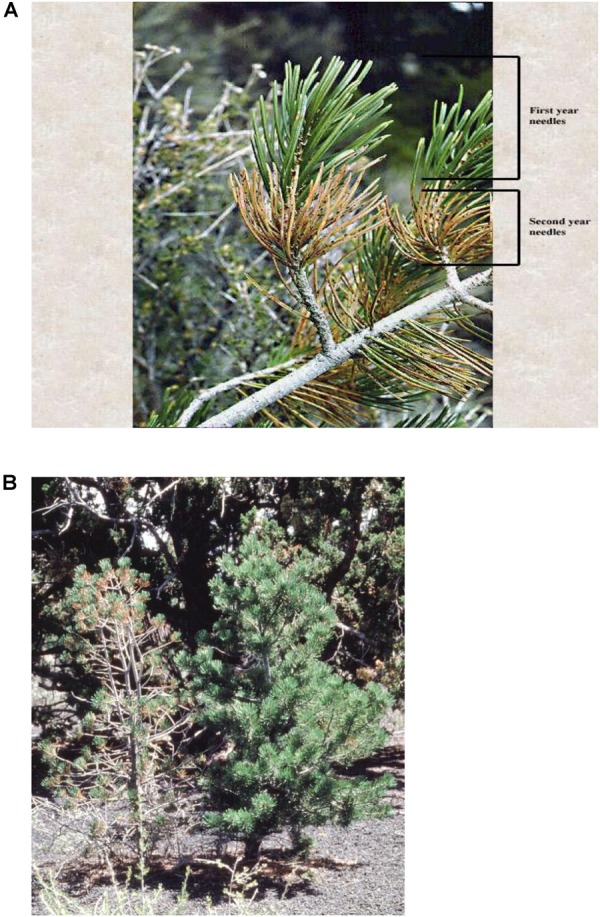
Pinyon needle scale attacks second year and older needle cohorts, but not the current year growth (scales are small black dots on chlorotic needles on the second year needles) **(A)**. Trees that are resistant and susceptible to pinyon grow side by side. On 1–2 m tall trees, scale densities on susceptible trees can exceed 500,000, while resistant trees with interdigitated branches may support 0 scales (Cobb and Whitham, 1993) **(B)**.

We quantified the arthropod community response to herbivory by the pinyon needle scale (*Matsucoccus acalyptus*) on juvenile pinyon pines that were resistant and susceptible to this insect. Scale herbivory on pinyon induces physical changes to susceptible trees and their local environment such as reduced foliage retention and mycorrhizal fungal colonization (Del Vecchio et al., 1993) and dramatically altered architecture (Cobb and Whitham, 1993). At high densities, scale herbivory causes extensive chlorosis and premature needle abscission (Figure 1A) leaving only the current year’s needles intact resulting in a “poodle tail” growth form (Cobb and Whitham, 1993) (Figure 1B). Increased needle senescence associated with susceptibility also alters micro-site characteristics such as soil temperature, litter decomposition, and nutrient cycling (Classen et al., 2005, 2007) and slows soil development (Classen et al., 2013).

Three lines of evidence argue that resistance and susceptibility to the scale insect is under genetic control. (1) Scale-infested trees with millions of scales commonly grow with their branches interdigitated with resistant trees with few or no scales (Figure 1B). (2) Long-term monitoring and tree ring analyses showed that patterns of scale infestation on individual trees in a stand remained consistent for 15 years such that susceptible trees support high scale loads (e.g., >6 scales per cm of needle) while resistant trees support few to no scales on the entire tree (Cobb and Whitham, 1993). Furthermore, these differences are reflected in the long-term tree ring record in which resistant trees produce rings 25% wider than susceptible trees (Trotter et al., 2002). Importantly, when scales are experimentally removed from susceptible trees, tree ring width rebounds to resemble resistant trees (Trotter et al., 2002). (3) Scale survival on resistant trees was only 9% compared to 50% on susceptible trees (Cobb and Whitham, 1993; Del Vecchio et al., 1993), and scales experimentally transferred onto resistant trees suffered 3-4X higher scale mortality than scales transferred onto susceptible trees (Gehring et al., 1997). When scales were transferred onto susceptible trees that had scales experimentally excluded for 8 years, scale survival did not differ from survivorship on the control susceptible trees despite differences in tree architecture (Gehring et al., 1997). In other words, long-term removal of scales resulted in a change in the architecture of susceptible trees to resemble resistant trees, but this did not alter their susceptibility to subsequent scale attack.

We predicted that indirect effects associated with changes in architecture and micro-site characteristics resulting from scale herbivory would affect other arthropod community members that reside on these trees. However, there might be direct genetic influences resulting from resistance and susceptibility to scales that also affect the associated community independent of the presence of scale insects. For example, differences in needle chemistry or resin production that may deter scale insects might also negatively impact other herbivorous insects. Because pinyon pine is habitat for a diverse arthropod community comprised of several hundred species (Trotter et al., 2008), differentiating between these two possibilities is important for understanding how a single genetically based interaction can influence higher-order diversity.

We sought to determine if arthropod community structure was more influenced by the indirect genetic influence of scale induced changes in tree architecture and associated traits, or the direct genetic influence of resistance and susceptibility acting on the associated community independent of the presence of scale. To separate the direct and indirect genetic influences of susceptibility to scale, we utilized an ongoing long-term scale exclusion experiment. For at least 15 years prior and throughout our study, scales were physically excluded from individual susceptible trees by removing the scale egg masses at the base of the tree (see Materials and Methods for more details). With long-term scale removal, these susceptible trees had recovered to exhibit the architectural phenotype of resistant trees (Figure 1B). By comparing resistant, susceptible and susceptible scale-excluded trees, we were able to separate the direct and indirect genetic effects of susceptibility to scale herbivory on the arthropod community.

Although direct and indirect influences of the scale-pinyon interaction on the arthropod community is genetic-based, the effects may not be consistent through time. Climate change is associated with declining insect biodiversity worldwide (Warren et al., 2018), and changes in climate are known to influence genetic-based interactions (Gehring et al., 2014a; Cooper et al., 2018). Indirect genetic effects under one set of environmental conditions might switch to direct genetic effects under another set of conditions. For example, community interactions that may depend more on foliage and habitat availability when environmental conditions are favorable, tree vigor is high, and communities are large and diverse may switch to be more dependent on tree genetics when drought conditions reduce both tree vigor and the overall richness and abundance of the arthropod community.

One predicted outcome of climate change in the southwestern US is an increase in the frequency and intensity of drought events (U.S. Global Change Research Program [USGRP], 2017). Pinyon pine is a foundation tree in this region that has been shown to be especially sensitive to drought (Breshears et al., 2005; Mueller et al., 2005; Gitlin et al., 2006). Because extreme climate events such as drought not only affect foundation tree species, but their associated arthropod communities, quantifying the effects of extreme drought on communities can add resolution to long-term predictions of the effects of climate change on arthropod diversity.

In this study, we sought to determine if the direct and/or indirect genetic influences of susceptibility to scale herbivory were influenced by climate by comparing arthropod community data on scale resistant and susceptible trees during an extreme drought year (2002), to data collected during a moderate drought year (2003) (NOAA/NCEI, 2018). We hypothesize that variation in drought intensity across years could impact both the direct and indirect genetic influence of pinyon on the arthropod community. If confirmed, such findings would suggest that interactions between climate and the genetic-based traits of foundation species are important to consider in understanding the effect of global change on biodiversity (Johnson and Agrawal, 2005; Gehring et al., 2017). Furthermore, arthropod richness and abundance were greatly depressed in the first year of the study due to extreme drought. By comparing those results to the subsequent moderate drought year we could address how resilient the arthropod community might be to drought stress. Particularly, we could determine if resistance or susceptibility to scale herbivory affects the ability of the arthropod community to recover from drought. The potential for community recovery from extreme environmental stress is likely to become a major issue as it is expected that with climate change, periods of record drought are likely to be interspersed with near normal or even wet periods (Cook et al., 2004; Gray et al., 2006).

## Materials and Methods

### Study Site and Drought Severity

Research was conducted near Sunset Crater National Monument in Flagstaff, AZ (elev. 2,000 m). The study area is co-dominated by pinyon pine (*Pinus edulis*) and one-seed juniper (*Juniperus monosperma*). Soils in the area consist of lava, ash, and cinders resulting in water limited and nutrient poor conditions (Gehring and Whitham, 1994; Cobb et al., 1997; Classen et al., 2005, 2007). Over the course of this study, northern Arizona experienced extreme drought conditions resulting in one of the hottest and driest summers on record in 2002. In 2003 the severity of the drought decreased, and this area received a 19% (29.57–35.26 cm) increase in annual precipitation which, coupled with slightly cooler temperatures caused the Palmer Drought Severity Index (PDSI) for northern Arizona to decrease from −4.08 to −2.55 (NOAA/NCEI, 2018). The PDSI is a region-specific index of meteorological drought calculated from precipitation, temperature and potential evapotranspiration (Palmer, 1965; Alley, 1984). The PDSI compares current year drought conditions to long-term average values and assigns positive values to above average wetter conditions and negative values to below average drought conditions. Because our study site fell on the border of two drought regions in Arizona (regions III and IV), we averaged the yearly PDSI values from these two regions in each year. Index values < −4 indicate extreme drought and index values between −3 and −2 indicate moderate drought conditions (Alley, 1984). Comparing these 2 years of differing drought intensity gave us the unique opportunity to quantify the impact of an extreme drought event on arthropod communities associated with pinyon, to assess the resiliency of the arthropod community, and to detect if there were interactions with genetic variation among the trees.

### Scale Lifecycle and Outbreak

Under conditions of nutrient and water stress, pinyon pine express resistance and susceptibility to a mesophyll feeding scale insect (*Matsucoccus acalyptus*). In the early spring, the scale insects emerge from egg clusters at the base of the trees and climb up to the branches where they attach themselves to the existing needles. Feeding usually begins in May and continues 6 months for males and 10 months for females (Cobb and Whitham, 1993). At high densities, scale feeding results in extensive chlorosis and premature needle abscission leading to an altered architecture where only the current year’s needles are retained (Cobb and Whitham, 1993) (Figure 1B).

Subsequent to a record drought in 1996, scale insects were able to expand their range out of small pockets of high nutrient and water stress in the ash and cinder fields around northern Arizona. In 1998 scales began to be observed along much of the western edge of the Colorado Plateau in the four corners region. This outbreak and range expansion was quantified by following the distribution of pinyon along the Mogollon rim and making observations at one-mile increments. Observational data was collected 1–3 times per year from 1990 to 1998. Pinyon were located by observations along major roads that followed pinyon-juniper woodland boundary lines as determined by U.S. Forest Service vegetation maps. Scale distribution boundaries were then further defined by using Forest Service roads to access trees. Where pinyon was observed, trees were driven or walked to until trees were encountered that had scales present on greater than 33% of their needles. Point observations of scale infested trees were cataloged with GPS and overlaid on the existing range of pinyon in ArcMap (version 8.0.1).

### Tree Selection and Sampling

To quantify the effect of extreme drought on the associated arthropod community on pinyon pine 60 trees were chosen from a group of trees associated with a long-term monitoring study which began in 1985. These trees were classified as resistant, susceptible or scale excluded at the time that monitoring began, and all trees used in this study had retained their original classification throughout the course of the monitoring period. Twenty trees from each of the three tree categories (scale susceptible, resistant, and scale excluded trees) were chosen for this study. Annual removal of scales began at the outset of the monitoring study in 1985 (Cobb and Whitham, 1993). Scales were completely removed from trees that supported high-density scale populations. Trees were defaunated by removing egg masses laid at the base of the trees in the early spring and Tanglefoot was placed approximately 5 cm above the ground to prevent any remaining scales from ascending the tree. The Tanglefoot was placed on duct tape so that it could later be removed in order to prevent other insects from potentially being affected. Tanglefoot was kept on the tree for several weeks to ensure that all of the eggs had hatched. Nearby non-treated trees were used to monitor when the eggs hatched. After hatching, egg masses turn from yellow to white as larvae move away from the eggs, leaving the white egg casings and filamentous fibers extruded by females behind. Within 10 years of having scales excluded in this way, scale excluded trees had similar amounts of foliage as scale resistant trees (Gehring et al., 1997). All three classes of trees were intermixed in an area of approximately 1 km^2^ and were juvenile pinyons between the ages of 40–60 years as determined by tree cores. The trees were blocked by height and basal trunk diameter to control for differences in tree age.

Due to the severity of the drought in 2002, only 16 of our 60 study trees survived over the winter. Because there were not enough surviving trees to do a repeated measures analysis, 20 new triplets were chosen in 2003 from the same study area that closely matched the height and basal trunk diameter of the original trees so that we could keep the age classes constant across years. These newly selected trees came from the same pool of trees that were associated with the long-term monitoring study. Like the first set of trees, the newly selected trees had been categorized as resistant, susceptible, or scale excluded when monitoring began in 1985 and had retained their original classifications throughout the period of this study. To address the hypothesis that the new trees selected from this pool might be physiologically different from the triplets used in the previous year, the 16 trees that survived both years were surveyed to compare their responses relative to the new trees.

To census arthropods, all branches on each tree were sampled using a muslin sweep net. The contents were emptied into sealable bags and stored in a freezer for subsequent analyses. Abundance, richness, and compositional data were recorded. In each year, arthropods were sampled over a 2-week period in late May in order to capture the greatest diversity within the community (Trotter et al., 2008; Stone et al., 2010). Because our sampling technique removed the arthropods from the tree, each tree was sampled only once during this period. All arthropods were categorized by morphotype and assembled into a reference collection that is stored at the Colorado Plateau Arthropod Museum at Northern Arizona University.

### Data Analysis

Richness and abundance data were analyzed using a blocked one-way analysis of variance (ANOVA) in the program JMPin (SAS, 2000; JMP 4.0). Trees were blocked by size across the three treatments to control for age differences among trees. Differences among treatment means were determined using Tukey HSD comparisons. To determine if there was an interaction between herbivore susceptibility and year-to-year variation in climate, a two-factor split plot design was implemented using the factors treatment (resistant, susceptible, scale excluded), year (2002, 2003), and the interaction between treatment and year.

For the 16 trees that survived both years, we plotted reaction norms for total arthropod richness and abundance on each tree to evaluate individual genotype responses to a reduction in drought intensity across years. Because these 16 trees were not included in the analyses of the newly selected trees in 2003, they represent an independent assessment of how drought interacts with resistance and susceptibility at the genotype level to affect the associated arthropod community. Differences in community composition were visualized using non-metric multidimensional scaling (NMDS) in the program PC-ord (McCune and Mefford, 1999). The community composition analysis was done with arthropod abundance relativized to species maximum. Relativization of the abundance data enabled us to equalize the importance of common and rare species in the analysis so that very abundant species would not overly influence differences among the groups. We did not include the scale insect in this analysis so that potential differences in community composition between susceptible and resistant trees would not be influenced by this one species. All pairwise comparisons among treatments were analyzed using a multi-response permutation procedure (MRPP) within PC-ord. Species accumulation curves were generated using EstimateS (Colwell, 2004). The species accumulation curves were generated according to Gotelli and Colwell (2001) using a randomized resampling technique of 1000 iterations with our samples defined as the individual trees from each of the three treatments.

## Results

### Scale Range Expansion With Drought

**FIGURE 2:**
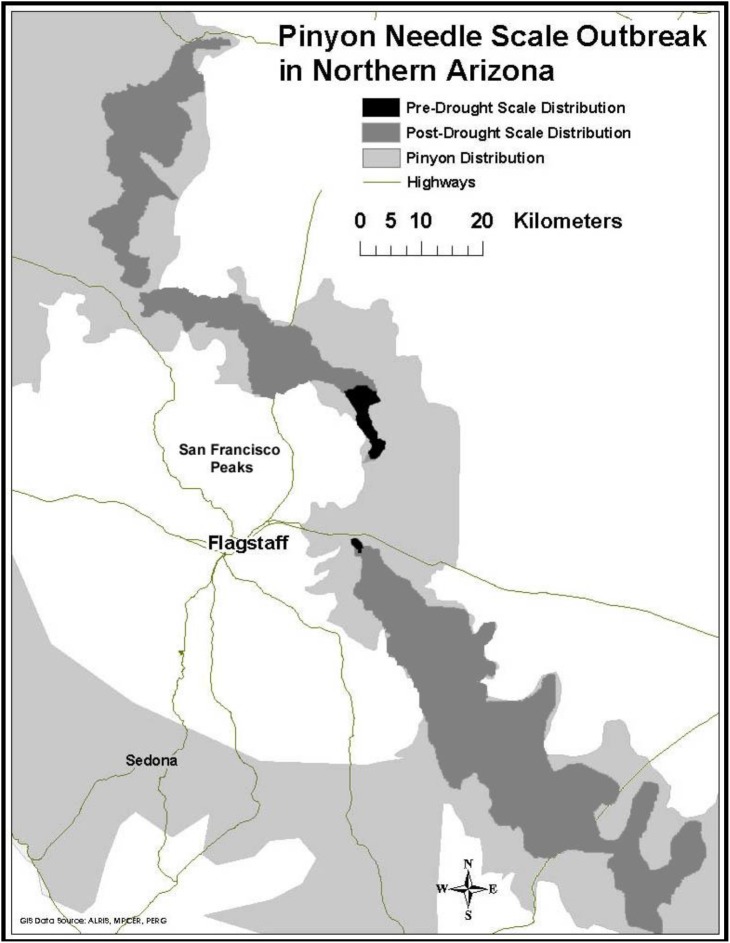
Range expansion of pinyon needle scale after a record drought in 1996. Dark gray represents pre-drought distribution of the scale insect, medium gray represents its post-drought distribution, and light gray represents the current distribution of pinyon pine.

Prior to 1996, our study area experienced above average rainfall in 16 of the previous 18 years (NOAA/NCEI, 2018). During this time scale insects were concentrated only in the high stress cinder soils associated with Sunset Crater National Monument. Starting in 1996 and continuing until the present time, this same region has experienced levels of drought ranging from abnormally dry to extreme (NOAA/NCEI, 2018). During this drought period scales have expanded their distribution into areas not formerly occupied (Figure 2). This range expansion with drought and the associated architectural changes associated with susceptibility has the potential to affect the associated community of arthropods representing several trophic levels.

### Scale Impacts on Associated Arthropod Community

**FIGURE 3:**
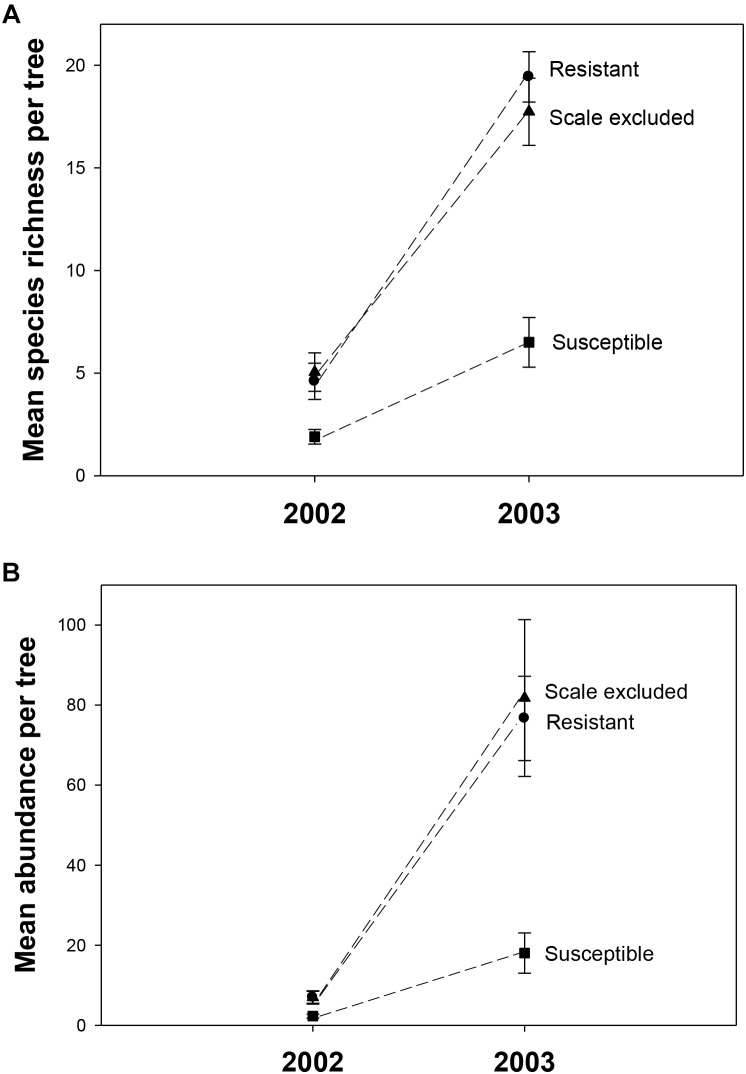
Mean species richness **(A)** and abundance **(B)** on scale excluded, scale resistant and scale susceptible trees in an extreme drought year (2002) and subsequent moderate drought year (2003). Dashed lines connect treatments between years.

In 2002, a total of 325 individuals comprising 90 morphospecies were collected. To determine if year-to-year variation in climate interacted with tree genetics we collected in the same manner in 2003. In 2003, 4046 individuals comprising 202 morphospecies from at least 30 families and 12 orders were collected. The number of families and orders are similar to another arthropod community study on pinyons in this area by Trotter et al. (2008), which found 287 species from 14 orders and 80 families. Across all treatments mean species richness per tree was 4X lower (3.8 ± 0.47 to 14.6 ± 0.99) and mean abundance was 11X lower (5.4 ± 0.80 to 58.8 ± 8.11) in the record drought year (2002) compared to the moderate drought year (2003). There was a consistent relationship across years in which susceptible trees had 2-3X lower richness (2002– *F* = 4.87, *p* = 0.01; 2003– *F* = 6.32, *p* = 0.016) and 3-4X lower abundance (2002– *F* = 4.15, *p* = 0.02; 2003– *F* = 18.16, *p* < 0.0001) than resistant and scale-excluded trees which did not differ from each other (Figure 3).

**FIGURE 4:**
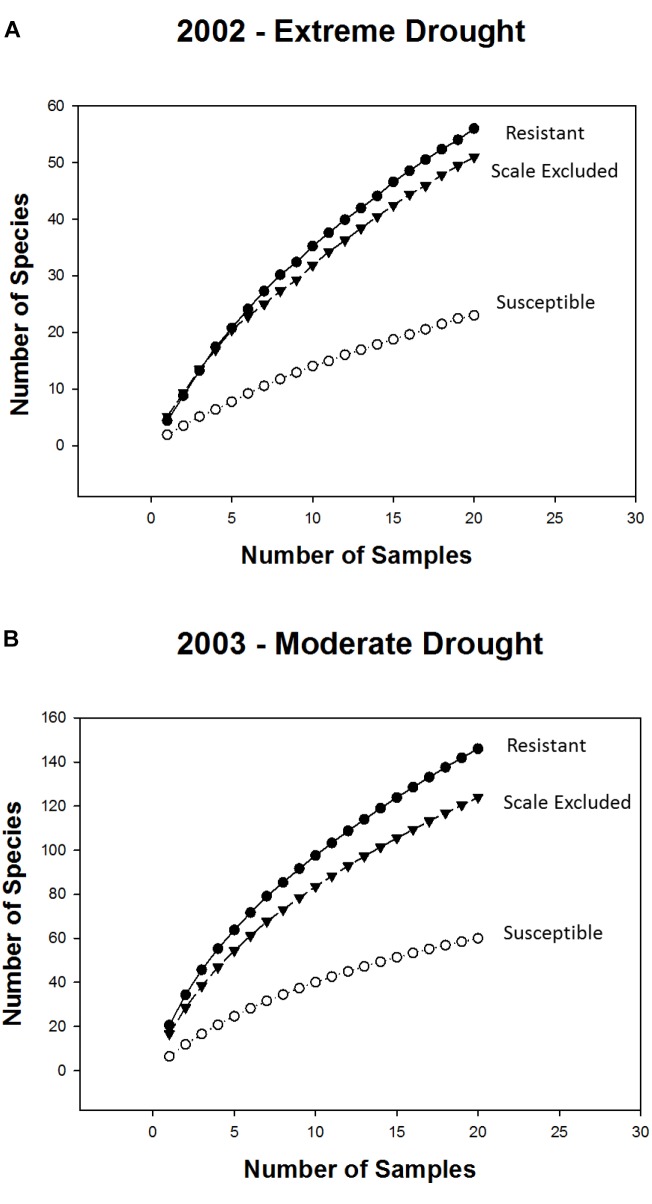
Sample based species accumulation curves for resistant, susceptible and scale excluded trees in the extreme drought year of 2002 **(A)** and the subsequent moderate drought year of 2003 **(B)**. Mean richness is represented by the first point of each curve. The rate at which new species appear in relation to sampling effort is represented by the shape of each curve where flatter curves indicate more consistent species composition across samples. Total richness is represented by the final point of each curve.

Consistent with the above findings species accumulation curves showed that, in both years, alpha diversity (mean richness, which is indicated by the first point of each curve) and gamma diversity (total richness, which is indicated by the final point on each curve) were higher on resistant and scale-excluded trees compared to susceptible trees (Figure 4). The accumulation curve for the susceptible trees was shallower, showing a rate of species accumulation that was much slower than either the scale-excluded or resistant trees.

#### Interactions Between Tree Genetics and Climate

Although there was consistency across years in the pattern of the response, we detected a significant interaction between treatment and year in which the differences in arthropod abundance (*p* = 0.007) and richness (*p* = 0.002) between resistant and susceptible trees was more pronounced in the moderate drought year compared to the extreme drought year (Table 1). Between 2002 and 2003 abundance increased 11X on resistant trees compared to 8X on susceptible trees while richness increased 4X on resistant trees compared to 3X on susceptible trees (Figure 3). These results demonstrate an interaction between tree genetics and climate in which the increase in richness and abundance associated with a decrease in drought severity is more pronounced on resistant trees compared to susceptible trees.

**Table 1:** Richness and abundance were significantly different among treatments in both years.

Source	SS	F-ratio	*p*-value
**Richness**			
Year	87.24	53.84	<0.0001
Treatment	44.90	34.11	<0.0001
Year × Treatment	9.22	7.01	0.002
**Abundance**			
Year	638.35	61.03	<0.0001
Treatment	207.74	13.51	<0.0001
Year × Treatment	82.89	5.39	0.007

*There was also a significant interaction between treatment and year in which the increase in richness and abundance between 2002 and 2003 was greater for resistant and scale excluded trees compared to susceptible trees.*

**FIGURE 5:**
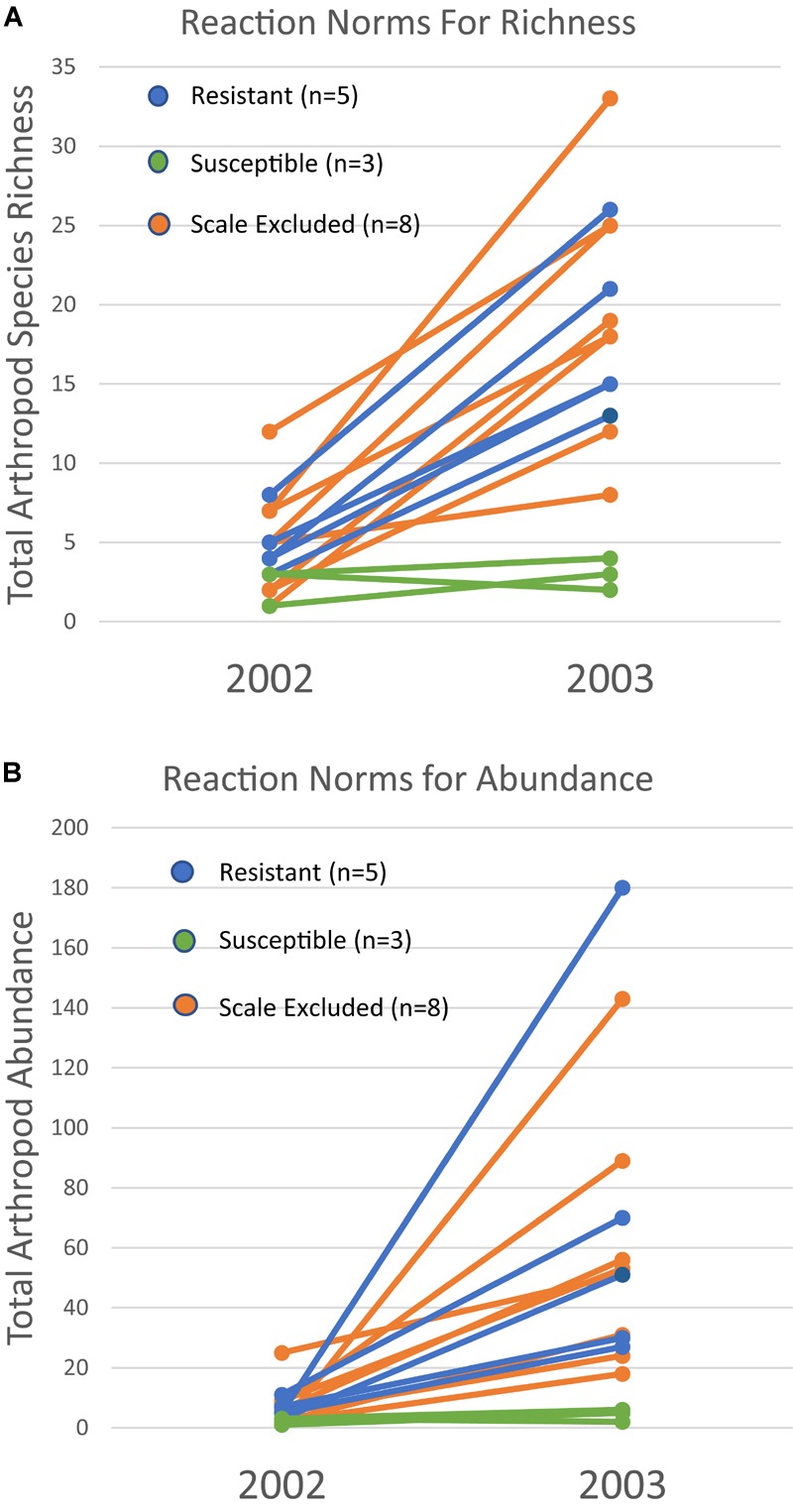
Reaction norms for 16 individual tree genotypes that survived both years of the study. Total species richness **(A)** and abundance **(B)** per tree show variation within and among susceptibility classes in their community plasticity responses to the extreme drought year of 2002 and moderate drought year of 2003. Each line represents the response of a single tree genotype across years.

Because a 73% mortality rate in our study trees prevented us from analyzing the same trees in both years at the treatment level, we had to choose a new set of 60 trees to use for the 2003 comparison. However, we did qualitatively examine the arthropod communities of the 16 surviving trees in 2003 to determine if patterns seen at the scale of individual tree genotype were similar to those seen at the treatment level in the newly selected trees. For the 16 surviving trees, reaction norms of total per tree species richness and abundance are plotted in Figure 5. These reaction norms show a more pronounced increase in both species richness and abundance on resistant and scale excluded trees compared to susceptible trees. These results are very similar to the findings at the treatment level (Figure 3) in that scale susceptible trees show a much reduced response to a reduction in drought stress. In addition, the reaction norms of individual tree genotypes show large tree-level variation in the response of the arthropod community to climate change. This indicates that some tree genotypes are more plastic in the way that their arthropod community responds to decreasing drought stress than other tree genotypes.

**FIGURE 6:**
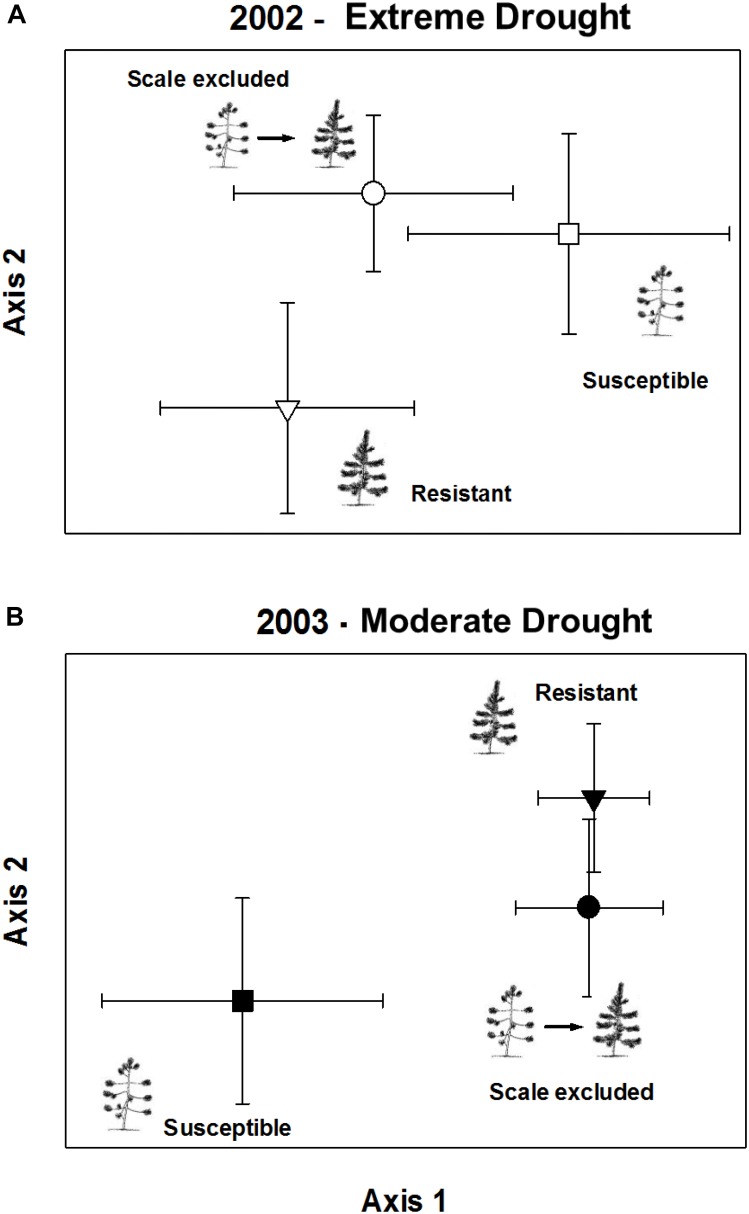
Non-metric multi-dimensional scaling ordination of arthropod community composition on scale excluded, scale resistant and scale susceptible trees in an extreme drought year 2002 **(A)** and subsequent moderate drought year 2003 **(B)**. Arthropod abundance is relativized to species maximum. Symbols represent the mean axis value of the community of arthropods on all trees within treatments ± 2 standard errors.

In the extreme drought year, comparisons of arthropod communities on resistant, susceptible, and scale-excluded trees indicated that the direct influence of scale susceptibility was driving arthropod community composition (Figure 6A). This is because the composition of the arthropod communities on scale-excluded trees did not differ from susceptible trees, and both were different from resistant trees (Chance-corrected within group agreement, *A* = 0.027; *p* = 0.013). However, in the subsequent moderate drought year, the pattern switched and composition of the arthropod community on scale-excluded trees no longer resembled the susceptible trees and did not differ from resistant trees (Chance-corrected within group agreement, *A* = 0.033; *p* = 0.0001) (Figure 6B). This demonstrates an interaction between tree genetics and climate that affects community composition. When the abundance of the arthropod community is low, traits related to tree architecture do not seem to have as much of an impact and the direct genetic influence of susceptibility to scale is more important in structuring communities. However, when the abundance of the arthropod community is high, factors related to tree architecture have a larger impact. Note that scale insects were not included in these analyses as our main goal was to examine how resistance and susceptibility affected the rest of the community independent of the scale insect.

## Discussion

### Indirect Genetic Influences of Susceptibility on Community Structure

The indirect influences of plants on community structure which are mediated through genetic-based interactions with herbivores have received increased attention (Werner and Peacor, 2003; Ohgushi et al., 2007; Keith et al., 2017), although most studies focus on chemically induced changes in plants (Denno et al., 1995; Van Zandt and Agrawal, 2004; Viswanathan et al., 2005) and not on morphological changes (but see Gehring et al., 1997; Crawford et al., 2007; Sthultz et al., 2009b). Susceptibility to scale affects both the resource quantity and the quality of pinyon pine by altering the tree’s architecture (Gehring et al., 1997; Chapman et al., 2003; Classen et al., 2005), as well as affecting microsite characteristics associated with individual trees such as soil moisture and temperature (Classen et al., 2005). We expected that morphological changes in pinyon such as a greater than 50% reduction in foliage (Gehring et al., 1997) would result in reduced food resources as well as increased exposure to predation that would deter many insects from using susceptible trees as habitat (Gonzalez-Megias and Gomez, 2003). This is supported by the result that in both years species richness was 2-3X higher, abundance was 3-4X higher and the composition of the community differed on resistant trees compared to susceptible trees (Figures 3, 6A). In addition, species accumulation curves demonstrate that susceptible trees accumulate new species much more slowly than resistant trees. In both years alpha diversity (mean richness) and gamma diversity (total richness) were all reduced on scale susceptible trees relative to resistant trees (Figure 4). This is in agreement with other research suggesting that morphological changes occurring as a consequence of susceptibility to herbivory can have as large of an impact on associated communities as physiological or chemical changes (Johnson and Agrawal, 2005). These findings also demonstrate that plant genetics can affect sampling effort as scale resistant trees require more sampling to characterize their communities relative to susceptible trees. If generally common, researchers may underestimate the importance of insect resistant trees in promoting biodiversity. Although our findings relate mainly to scale-altered architecture (e.g., a single cohort of needles rather than 6+ years of needle cohorts), it is important to note that plant phytochemistry, phenology, nutrient availability and other important plant traits could also be affected by susceptibility to the scale insect, which deserve future study.

### Direct Genetic Influences of Susceptibility on Community Structure

Many differences in arthropod community structure between resistant and susceptible trees are based on the indirect influence of susceptibility mediated by a change in tree architecture and associated traits. However, it is likely that there are also direct influences on community structure that stem from genetic differences related to resistance and susceptibility. To separate the indirect influence of architectural changes induced by scale insects from the direct influence of tree genetics we utilized a scale exclusion treatment in which scales were experimentally excluded from susceptible trees for 15 years. Excluding scales for this amount of time allowed the tree architecture of the susceptible trees to grow back 6+ years of needle cohorts to resemble resistant tree architecture. When we compared scale-excluded susceptible trees to resistant and scale-altered susceptible trees we found that richness, abundance, and the accumulation of species did not differ between resistant and scale-excluded susceptible trees but both were significantly different from scale-altered susceptible trees. This pattern was observed in both the extreme drought year of 2002 and the subsequent moderate drought year of 2003. These results argue that metrics such as arthropod richness and abundance are more tied to the morphology of the trees and that arthropods are responding, for example, to the amount of foliage as a resource for factors such as food or predator avoidance. These findings are in agreement with other studies that have found that morphological changes associated with herbivory are more important in structuring communities than the direct influence of tree genetics (i.e., susceptibility to scales might also indicate susceptibility to other arthropods). Dickson and Whitham (1996) showed that with the removal of the galling aphid, *Pemphigus betae*, from susceptible cottonwood trees, species richness and abundance declined to resemble resistant trees, arguing that the plant genetic effects on the community acted indirectly (i.e., through the presence of aphid galls) rather than directly on the rest of the community. However, our studies are different in that we found that the presence of scales negatively affected species richness and abundance, whereas Dickson and Whitham (1996) found that the aphid positively affected species richness and abundance. This difference is likely because the galling aphids provided food for predators and parasitoids, did not severely damage their hosts, and created habitat for inquiline species that used the galls as habitat (Martinsen et al., 2000). Scale insects, however, damage their host plants causing most needles to prematurely abscise thereby eliminating resources for other arthropods (Cobb and Whitham, 1993; Trotter et al., 2002).

When we compared the composition of the arthropod communities among the three treatment groups in the extreme drought year of 2002, we found that the composition of the community on scale-excluded trees differed from that of resistant trees but was not significantly different from susceptible trees. This result argues that metrics such as richness and abundance are more influenced by tree architecture and associated traits, whereas the composition of the community is more directly influenced by tree genetics. Certain arthropod species were more likely to be found on the susceptible genotype whether or not the scale insect was present. Despite large differences in tree architecture, richness, and abundance between scale-excluded and scale-altered susceptible trees, they had similar community compositions. This is in agreement with Wimp et al. (2005) who found that on two species of cottonwoods and their F_1_ hybrids, species richness and abundance did not differ among the groups, but they all had unique community composition. The findings of Wimp et al. (2005) and this study suggest that because of the complex interactions that create community structure, genetic influences that directly impact even a few species can cascade up to create large differences in the composition of the whole community.

### Interactions Between Tree Genetics and Climate

From 2002 to 2003 total species richness of the arthropod communities on pinyon quadrupled and abundance increased 11-fold. This corresponded with a decrease in drought severity from extreme to moderate drought. While this result shows remarkable resilience in the arthropod community, this resilience was not similarly experienced by resistant and susceptible trees. Although arthropod richness and abundance increased on both resistant and susceptible trees from 2002 to 2003, there was a treatment × year interaction in which the increase in both richness and abundance was greater on resistant trees compared to susceptible trees (Table 1). Although the arthropod community has the potential to rebound on both resistant and susceptible trees after an extreme drought event, the increase in richness and abundance on susceptible trees did not keep pace with resistant trees. The resilience of the arthropod community after drought is impeded by the genetic-based interaction between pinyon and scales. It is important to consider how different community responses to stress (i.e., linear versus non-linear) might affect these findings. For example, if richness and abundance increased exponentially rather than linearly, what would appear to be a treatment by year interaction in a linear model might be explained by a different rate of increase in richness and abundance on resistant and susceptible trees along an exponential curve. Regardless, genetic influences that affect even just the rate of recolonization of species can be important to the resiliency of the arthropod community.

The patterns demonstrated with resistant, susceptible and scale excluded trees at the treatment level (Figure 3) are also reflected in our plots of the reaction norms of 16 individual tree genotypes that survived both years (Figure 5). These reaction norms show that individual tree genotypes within treatments differ greatly in their response to drought severity, and that even within a treatment reaction norms can be different. These community-level reaction norm findings are similar to those observed by Keith et al. (2017) who found significant within group variation of arthropod richness and abundance on cottonwood genotypes with aphids and with aphids excluded in a common garden. Because the arthropod community associated with pinyon participates in many trophic interactions across multiple taxa, our findings argue that direct genetic-based interactions such as between pinyon and the scale insect have important community-level effects. To our knowledge, no other study has demonstrated the impact of a fundamental genetic-based plant-herbivore interaction on the resiliency of the associated arthropod community after extreme drought, but these interactions are important to consider when predicting the impacts of climate change on biodiversity.

These community level effects have been shown to be associated with large ecosystem-level effects as well. For example, studies by Classen et al. (2006) at the same study site showed that scales significantly increase needle litter nitrogen (N) and phosphorus (P) concentrations by 50%, as well as litter inputs to soil by 21%. In addition, microbial biomass was 80% lower in soils beneath scale-susceptible trees when compared to resistant trees. Classen et al. (2005) also showed that scale herbivory reduced leaf area index (LAI) of susceptible trees by 39% and that scale herbivory increased soil moisture and temperature beneath susceptible trees by 35 and 26%, respectively. The authors concluded that the magnitude of scale effects on soil moisture and temperature is similar to global change scenarios, and sufficient to drive changes in ecosystem processes that ultimately slow soil development (Classen et al., 2013). Since the expression of susceptibility is mediated by climate, the expansion of scale insects with drought (Figure 2) is likely to have important ecosystem-level consequences.

In the extreme drought year community composition on scale-excluded susceptible trees did not differ from scale-altered susceptible trees and both were different from resistant trees. However, in the subsequent moderate drought year when environmental conditions improved and the arthropod community rebounded, the pattern switched and the composition of the community on scale-excluded susceptible trees became similar to resistant trees and both were different from susceptible trees (Figure 6). Under poor environmental conditions such as the record drought of 2002 when trees suffered very high stress, the abundance of the arthropod community was very low. With low abundance, we hypothesize that extrinsic factors such as decreased interactions among the arthropods may make it more likely that they respond to the genetics of the trees and we find similar communities on susceptible genotypes (with and without scales) regardless of their architecture. However, when conditions improve and arthropod abundance and richness increase, traits associated with tree architecture become more important in structuring communities.

Our findings link the effects of climatic stress with genetic-based susceptibility to herbivory and show a strong negative impact on the diversity and resilience of arthropod communities. This study fits in to a broader context of community-level research which deals with the effects of environmental stress on arthropod communities. Within a stand, Stone et al. (2010) found that insect diversity is greatest on vigorously growing pinyons and declines to near zero on stressed trees exhibiting slow growth, branch dieback and high needle abscission. Similarly, across stands varying in soil type, Trotter et al. (2008) found that trees growing under high stress in cinder soils supported about 1/10th the number of arthropods, and roughly half the species as trees growing under more favorable conditions in sandy-loam soils. The median ratio of herbivores to predators at low stress sites was 17.8:1 while in the high stress sites the ratio dropped to 1.7:1. This order-of-magnitude shift herbivore-predator ratio indicates both a change in community composition and trophic structure in which predator pressures on herbivores may dramatically increase in stressed environments. Thus, at multiple levels of study (i.e., genetic differences among trees within a stand in their susceptibility to a common herbivore, among trees within a stand that differ in tree vigor, and across stands that differ in stress levels associated with different soil types), chronic herbivory, climatic stress, and soil stressors each have similar negative impacts on arthropod community metrics. All three of these stressors combine to impact scale susceptible trees growing in poor soils during drought years. To understand the effects of global change on arthropod communities, we need to integrate the impacts of multiple and simultaneously occurring biotic and abiotic stressors that often occur together. To better understand these observationally and experimentally derived patterns, more mechanistic studies are needed.

These results are among the first to demonstrate tree genetics interacting with climate to affect the richness, abundance, and composition of arthropod communities. The results of this study demonstrate that climatic changes have the potential to alter fundamental genetic-based interactions between foundation tree species and their associated arthropod communities (Maddox and Cappuccino, 1986; Gilman et al., 2010). We found that the arthropod community response to both the direct genetic influence of resistance and susceptibility to scale and the indirect genetic influence of susceptibility manifested in altered tree architecture can be influenced by climatic changes that occur over the span of a single year. We are aware of only one study that has utilized an herbivore removal experiment to separate direct and indirect genetic influences of a foundation tree on its dependent community under two different environmental conditions to determine if the community response remained the same. Sthultz et al. (2009b) found that the composition of ectomycorrhizal fungal (EMF) communities on mature pinyon pine (*Pinus edulis*) were very different on trees that were resistant to the stem and cone-boring moth (*Dioryctria albovittella*) and trees that were susceptible. Susceptible trees exhibit a more shrub-like and closed architecture, but long-term removal of moths from susceptible trees allowed the susceptible trees to recover and resemble the architecture of resistant trees. They found that during both wet and drought years, plant genetic effects on EMF community composition were direct. In other words, plant genetics was more important than tree architecture in determining EMF community composition across both a drought year and a wet year. Our results do not show the same consistent pattern across climatically variable years. This could be because mycorrhizal fungi are generally mutualists, whereas arthropod communities generally contain many antagonistic herbivores. Furthermore, mycorrhizal fungi are physically attached to their host, while most arthropods are mobile. The direct interactions between a host tree and the mycorrhizal fungal community compared to the arthropod community are fundamentally different and are likely to be affected differently by changes in tree condition in response to environmental change. Regardless, if climate change alters fundamental genetic-based interactions of some species but not others, then it becomes especially important to understand and predict which interactions might be most sensitive to climate change.

In their review of community and ecosystem genetics, Whitham et al. (2006) argued that changes in global climate will create new selection pressures on foundation species that can affect the evolution of associated communities. Current predicted patterns of climate change involve not only increasing mean temperatures, but increased year-to-year variation in weather patterns and more frequent extreme climatic events such as drought (U.S. Global Change Research Program [USGRP], 2017). Recent climate change studies show that the American southwest is undergoing severe drought (U.S. Global Change Research Program [USGRP], 2017) that is resulting in widespread mortality of foundation tree species (Breshears et al., 2005; Gitlin et al., 2006; van Mantgem et al., 2009; Whitham et al., 2019). In 2002 the southwest experienced drought of an intensity that had not been observed in 50 years (Breshears et al., 2005). Although droughts of this severity are rare, they provide unique opportunities to quantify short-term ecological responses to extreme events (Holmgren et al., 2001; Reusch et al., 2005; Gehring et al., 2014b). Quantifying the impact of drought and the biotic response are especially important because the intensity and frequency of drought in this region is predicted to increase (Seager et al., 2007; U.S. Global Change Research Program [USGRP], 2017), which is likely to result in major shifts in the geographic distribution of many foundation tree species (Parmesan, 2006; Rehfeldt et al., 2006; O’Neill et al., 2008; Ikeda et al., 2017), their associated communities (Gilman et al., 2010) and outbreaks of herbivores such as bark beetles which attack trees that have compromised defenses due to environmental stressors (Raffa et al., 2008). Mortality and range shifts in these important tree species due to drought is worldwide (Allen et al., 2010) and likely to have major consequences for the communities they support (Ikeda et al., 2014). Because the expression of susceptibility is often stress dependent (i.e., in the absence of climate stress all trees appear to be resistant, but with stress, susceptible and resistant phenotypes become apparent) (Cobb et al., 1997) (Figure 2), and additional outbreaks may be more frequent with increasing drought, our findings suggest that climate change in the southwest is likely to result in a substantial loss of arthropod diversity at a regional scale.

## Author Contributions

AS designed the study, collected and analyzed the data, wrote the manuscript. NC, CG, and TW initiated the scale removal experiment, helped design the study, and revised the manuscript.

## Conflict of Interest Statement

The authors declare that the research was conducted in the absence of any commercial or financial relationships that could be construed as a potential conflict of interest.
